# Itaconate family-based host-directed therapeutics for infections

**DOI:** 10.3389/fimmu.2023.1203756

**Published:** 2023-05-16

**Authors:** Jae-Min Yuk, Eun-Jin Park, In Soo Kim, Eun-Kyeong Jo

**Affiliations:** ^1^ Infection Control Convergence Research Center, College of Medicine, Chungnam National University, Daejeon, Republic of Korea; ^2^ Department of Medical Science, College of Medicine, Chungnam National University, Daejeon, Republic of Korea; ^3^ Department of Infection Biology, College of Medicine, Chungnam National University, Daejeon, Republic of Korea; ^4^ Department of Microbiology, College of Medicine, Chungnam National University, Daejeon, Republic of Korea; ^5^ Department of Pharmacology, College of Medicine, Chungnam National University, Daejeon, Republic of Korea

**Keywords:** itaconate, innate immunity, toll-like receptor, inflammation, host defense

## Abstract

Itaconate is a crucial anti-infective and anti-inflammatory immunometabolite that accumulates upon disruption of the Krebs cycle in effector macrophages undergoing inflammatory stress. Esterified derivatives of itaconate (4-octyl itaconate and dimethyl itaconate) and its isomers (mesaconate and citraconate) are promising candidate drugs for inflammation and infection. Several itaconate family members participate in host defense, immune and metabolic modulation, and amelioration of infection, although opposite effects have also been reported. However, the precise mechanisms by which itaconate and its family members exert its effects are not fully understood. In addition, contradictory results in different experimental settings and a lack of clinical data make it difficult to draw definitive conclusions about the therapeutic potential of itaconate. Here we review how the immune response gene 1-itaconate pathway is activated during infection and its role in host defense and pathogenesis in a context-dependent manner. Certain pathogens can use itaconate to establish infections. Finally, we briefly discuss the major mechanisms by which itaconate family members exert antimicrobial effects. To thoroughly comprehend how itaconate exerts its anti-inflammatory and antimicrobial effects, additional research on the actual mechanism of action is necessary. This review examines the current state of itaconate research in infection and identifies the key challenges and opportunities for future research in this field.

## 1 Introduction

Innate immune responses are the primary host defense to infection. Macrophages participate in innate immunity by recognizing pathogen- or danger-associated molecular patterns. Upon activation, macrophages initiate an intracellular signaling program to activate the expression of numerous genes involved in inflammatory, immune, and antimicrobial responses. Simultaneously, innate immune cells undergo significant metabolic reprogramming depending on their differentiation status (1–3). The metabolites up- or down-regulated during infection act as signals to modulate immune pathways, antimicrobial responses, and homeostasis (4–6).

**Figure 1:**
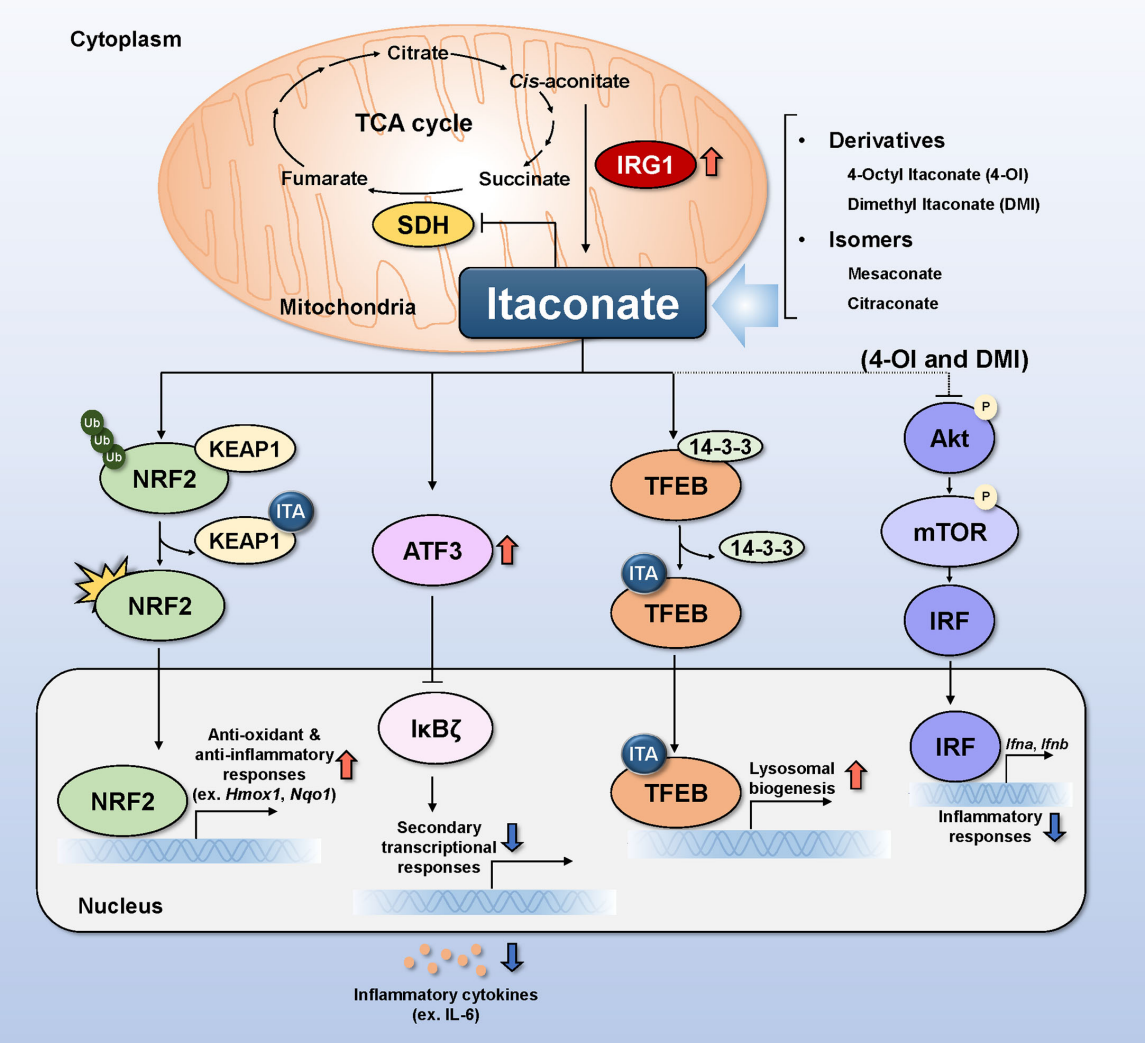
Molecular mechanisms of itaconate and its relatives in infection. Itaconate (ITA) is produced from the decarboxylation of *cis*-aconitate in mitochondria in response to IRG1 induction. Itaconate modulates the activity of SDH by competing with succinate, thereby regulating the TCA cycle. Itaconate causes KEAP1 to dissociate from the NRF2-KEAP1 complex by alkylating cysteine residues 151,257,288,273, and 297 on KEAP1. Translocation of activated NRF2 to the nucleus increases anti-oxidant and anti-inflammatory gene expression. ATF3 induced by itaconate translocates to the nucleus to inhibit IkBζ, thereby preventing the production and secretion of inflammatory cytokines. TFEB alkylated by itaconate on cysteine residue 212 elicits translocation to the cell nucleus, resulting in the upregulation of genes related to lysosomal biogenesis. DMI and 4-OI decrease Akt phosphorylation, whereas ITA increases it. Inhibiting Akt phosphorylation inhibits mTOR/IRF signaling and the production of type I interferon (*Ifna* and *Ifnb*). Alk, alkylation; ATF3, activating transcription factor 3; IκBζ, IkappaB-zeta; IL, interleukin; IRF, interferon-regulated factor; IRG1, immune-responsive gene 1; KEAP1, Kelch ECH associating protein 1; NF-κB, nuclear factor-kappa B; NRF2, nuclear factor erythroid 2-related factor 2; SDH, succinate dehydrogenase; TFEB, transcription factor EB.

Itaconate (ITA), a signaling metabolite produced by classically activated macrophages (7), regulates the immune, inflammatory, and oxidative responses to infection (8–10). The intrinsic pathway of endogenous ITA production in macrophages requires immune-responsive gene 1 (IRG1), also known as aconitate decarboxylase 1 (ACOD1), to decarboxylate *cis*-aconitate (**Figure 1**) (11). Normally, *cis*-aconitate does not dissociate from aconitase, the enzyme catalyzing the dehydration of both citrate and isocitrate, and at equilibrium, the substrates of aconitase are present 90% of citrate, 6% of isocitrate, and 4% of cis-aconitate (12–14). The expression of *Irg1* encoding ACOD1 shows basal level in nonactivated macrophages, though the gene level is induced upon infection with live pathogens or LPS stimulation (11, 15). Classically activated M1 macrophages undergo dynamic immunometabolic remodeling, manifesting as early accumulation of succinate and ITA, during infection and inflammation, and the accumulation of the two molecules is correlated with each other (7, 16). ITA inhibits succinate dehydrogenase (SDH) competitively based on structural similarity with succinate (**Figure 1**) (17, 18).

To circumvent the low plasma membrane permeability, ITA is esterified and investigated as 4-octyl ITA (4-OI) or dimethyl ITA (DMI) (17, 19, 20). ITA, its esterified derivatives (4-OI and DMI), and its naturally occurring isomers (mesaconate and citraconate) make considerable contributions to infectious and inflammatory diseases. Indeed, the anti-infective and anti-inflammatory roles of ITA and its isomers and esterified derivatives have been discussed (8–10, 21–23). Here, we review the regulation of endogenous ITA production in terms of immunometabolic networks and the functions of ITA and its relatives during infection. We also focus on the molecular mechanisms by which ITA and its related members regulate innate and inflammatory responses in infection and immunity.

## 2 Immune regulation *via* the IRG1-ITA pathway during infection

Classical activation of macrophages toward the M1 phenotype drives metabolic reprogramming, leading to upregulated glycolysis, disruption of the TCA cycle, and ITA accumulation (24). In *Mycobacterium tuberculosis* (Mtb) infection, the metabolite glutamine drives M1 macrophage responses *via* immunometabolic remodeling in which the biosynthetic precursor ITA is generated (25). In turn, ITA functions as a feedback inhibitory regulator by TCA-cycle reprogramming in macrophages (26). That is, ITA inhibits isocitrate dehydrogenase 2 (IDH2), thereby altering the mitochondrial NADP+/NADPH ratio and inhibiting SDH (26). In addition, interleukin (IL)-33-mediated metabolic rewiring in macrophages upregulates ITA production, and ITA promotes the GATA3-mediated polarization of alternatively activated macrophages, thereby contributing to tissue repair and the resolution of inflammation (27). Moreover, 4-OI suppresses aerobic glycolysis by directly alkylating Cys22 of GAPDH, thus inhibiting inflammatory responses in activated macrophages (28).

Macrophage stimulation by toll-like receptor (TLR) ligands activates ITA production (29). In human monocytic THP-1 cells, lipopolysaccharide (LPS) stimulation upregulates IRG1 mRNA *via* cyclin-dependent kinase 2 (CDK2)-mediated JUN activation and IRG1 accumulation, thereby robustly activating the pro-inflammatory tumor necrosis factor-α (TNF-α) signaling pathway (30). In addition, the host TLR2, myeloid differentiation primary response 88 (MyD88), nuclear factor-kappa B (NF-κB), stimulator of interferon genes (STING), and type I interferon (IFN) receptor signaling pathways induce IRG1 expression during Mtb infection (31). Signals from phagocytosis and endosomal acidification are needed to induce IRG1 expression in bone marrow-derived macrophages (BMDMs) (31). In *Brucella* infection, MyD88 signaling is required for ITA production and ITA-mediated antibacterial responses to *B. melitensis* in macrophages (32). By contrast, IRG1-mediated ITA production is suppressed by the induction of β-glucan-mediated trained immunity, thus modulating immunoparalysis during sepsis (33).

Type I and II IFNs trigger the expression of IRG1 and ITA to exert bactericidal functions against *Legionella pneumophila*, and extracellular multidrug-resistant gram-positive and negative bacteria (34). The pro-inflammatory cytokines TNF-α and IL-6 activate ITA-mediated direct antimicrobial responses in *M. avium*-infected macrophages (35). TNF-α and IL-6 activate paracrine signaling to promote the IRF1/IRG1 pathway and the repositioning of mitochondrial to bacterial phagosomes during *M. avium* infection (35). Therefore, the inflammatory responses of bystander cells at infection sites may contribute to endogenous ITA production, thereby amplifying antimicrobial responses during infection.

## 3 Roles of ITA in infection

### 3.1 ITA-induced protection

In most infection models, ITA and its family members are considered antimicrobial metabolites, because they target isocitrate lyase of the glyoxylate shunt during *Salmonella enterica* and Mtb infections (11). *In vivo*, the IRG1-ITA pathway ameliorates neutrophil-mediated pathologic inflammation to promote antimicrobial responses against Mtb infection (36). Also, the endogenous ITA-mediated restriction of intracellular bacteria such as *S.* Typhimurium depends on the guanosine triphosphatase Rab32, which interacts with IRG1 to deliver the antimicrobial factor ITA to the *Salmonella*-containing vacuole (37). In addition, the T helper cell 1 (Th1)-induced cytokine IFN-γ stimulates the production of ITA, which inhibits mitochondrial complex II to increase bactericidal activity against *Francisella tularensis* (38).

*Brucella* infection upregulates IRG1, which is critical for the control of *Brucella* growth, in murine alveolar macrophages (39). Notably, ITA and DMI exhibit direct antimicrobial effects against *Brucella* by targeting isocitrate lyase of *B. abortus* (39). Similarly, in a *Vibrio* infection model, ITA suppresses the growth of *Vibrio* sp. DO1 (40). Moreover, ITA reduces intracellular *Escherichia coli* at later time points in macrophages, at least in part by increasing phagocytosis and bactericidal activity (41). In addition, DMI suppresses intracellular growth of Mtb, *M. avium*, even of multidrug resistant Mtb in macrophages, partly associated with the induction of autophagy (22). In Zika virus infection of neurons, receptor interacting protein kinase 1 (RIPK1) and RIPK3 signaling suppresses viral replication *via* IRG1-mediated ITA production (42). In addition, IRG1 is essential for the restriction of *Coxiella burnetii* infection, which causes zoonotic Q fever, in macrophages and intratracheal or intraperitoneal infection models. IRG1 deficiency amplifies inflammatory responses—including the expression of *Il6*, *Ifng*, *Nos2*, and *Gbp1*—in the lungs of infected mice. Interestingly, exogenous ITA reduces the bacterial burden, and the physiologic concentration of ITA is sufficient to control *C. burnetii* replication (43). Furthermore, in chronic infection with *Toxoplasma gondii*, which impairs cognitive functions, treatment of infected mice with DMI improves behavioral performance and ameliorates microglial inflammation (44).

There are few reports on the clinical relevance of ITA in human infectious diseases. Interestingly, multidrug-resistant tuberculosis (TB) patients show an inflammatory metabolic response, which manifests as upregulated succinate and downregulated ITA, which is increased in patients on appropriate anti-TB treatment (45). Therefore, host metabolic remodeling accompanied by decreased ITA drives immunopathological responses in human TB.

### 3.2 ITA pathological functions

Some findings indicate a pathologic or insufficiently protective role for the IRG1-ITA pathway in infection. Respiratory syncytial virus (RSV) infection triggers IRG1 expression to promote reactive oxygen species (ROS) generation in human A549 cells, immune cell infiltration, and lung injury *in vivo* (46). In addition, the dysfunctional complex of phosphatase and tensin homolog deleted on chromosome 10 (PTEN) with the cystic fibrosis (CF) transmembrane conductance regulator (CFTR), which is associated with the pathogenesis of cystic fibrosis, increases the production of succinate and IRG1-ITA (47). Nevertheless, these metabolic changes are not sufficient to clear *Pseudomonas aeruginosa* due to impaired PTEN activity and excessive oxidative stress (47). IRG1 and ITA are required for bacterial persistence and host tolerance during infection with *Klebsiella pneumoniae* sequence type 258 (Kp ST258) (48). Kp ST258 infection drives host metabolic pathways towards glutaminolysis, fatty acid oxidation, and accumulation of ITA, resulting in anti-inflammatory M2-type responses and disease-tolerant immune responses (48). Together, these recent studies raise the question of how the IRG1-ITA pathway contributes to host detrimental responses rather than protection in certain types of infection.

Intracellular microbes and parasites can distort the IRG1/ITA axis and use ITA during infection (44). Influenza A virus (IAV) infection increases *Irg1* mRNA expression in M2-type human macrophages and undifferentiated peripheral blood mononuclear cells (PBMCs) (49). In a rabbit model of *P. aeruginosa* (PAO1) infection, IRG1 induction and ITA production in host cells may contribute to bacterial adaptation and biofilm formation by enabling use of ITA as a carbon source in the acute phase of wound infection (49). Some bacteria such as *P. aeruginosa* clinical isolates can establish infection and replicate in host cells by using ITA as their major carbon source (50). Upon exposure to ITA, *P. aeruginosa* produces extracellular polysaccharides (EPS), which stimulate the production of ITA in host cells (50). In addition, ITA inhibits glycolysis in *Staphylococcus aureus*, a pathogen easily adaptable to the host immunometabolic environment, and increases the synthesis of extracellular polysaccharide and biofilm formation (51). Furthermore, in a vaccine model of *Francisella tularensis* infection, *Irg1* deficiency increases resistance to secondary challenge by promoting CD4+ and CD8+ T cell responses (52). Together, these results suggest that pathogens can use endogenous ITA as a nutrient to establish persistent infection by modulating host immune pathways. Further studies are needed to determine how pathogens manipulate the IRG1-ITA axis to influence innate and adaptive immune pathways.

## 4 Mechanisms by which ITA and its family members control infection

There are several mechanisms by which ITA and its family members exert antimicrobial effects during infection; we briefly discuss the four major mechanisms, i.e., nuclear factor erythroid 2-related factor 2 (NRF2), activating transcription factor 3 (ATF3), transcription factor EB (TFEB), and Akt, below. And these are summarized in **Figure 1** and **Table 1**.

**Table 1:** Host protective functions of itaconate and its relatives as therapeutic candidates in infectious/inflammatory diseases.

Type of ITA	Infectious agents	Models	Functions	Ref
Intermediated by NRF2				
**DMI**	*A. fumigatus*	*In vivo*: - Fungal keratitis model *In vitro*: - Human corneal epithelial cells	Host protection against fungal keratitis - ↓ Clinical scores, PMN infiltration, and fungal load in eyes of mice - ↓ Inflammatory responses in response to fungal keratitis - ↓ IL-1β and CXCL1 in HCECs - ↓ IL-1β, IL-8, and IL-6 in infected corneas Activation of Nrf2/HO-1 signaling pathway - ↑ Nrf2 and/HO-1 expression in DI-treated corneas of mice - ↑ Nuclear Nrf2 accumulation in HCECs	(53)
	LPS	*In vivo*: - LPS-induced septic model *In vitro*: - BMDMs	Host protection against LPS-induced inflammation - ↓ Mice lethality and inflammation score in LPS-induced septic models - ↓ LPS-Induced production of inflammatory cytokines in BMDMs - ↓ IL-1β and CXCL1 in HCECs · Activation of Nrf2 signaling pathway - ↑ Level of Nrf2 and its target genes HO-1 and NQO1 in both LPS-treated mice and murine macrophages - No effects in LPS-treated *Nrf2* ^-/-^ mice.	(54)
**4-OI**	LPS	*In vivo*: - CLP-induced septic model *In vitro*: - RAW 264.7	Host protection against septic model - ↓ Mice lethality, tissue injury, and inflammation score Negative regulation of LPS-induced inflammation in RAW 264.7 cells - ↓ M1 and ↑ M2 polarization - ↓ IFN-γ, IL-1β, TNF-α expression or ROS release - ↑ IL-10 secretion Activation of Nrf2/PD-L1 signaling pathway - ↑ Nrf2 gene transcription and protein expression - ↓ LPS-induced oxidative stress and PD-L1 *via* Nrf2 signaling	(55)
	SARS-CoV2 HSV1, 2 VACA Zika virus	Patient sample: - COVID-19 biopsies *In vitro*: - Vero cells - Calu-3 - NuLi cells - A549 cells - HaCaT - PBMCs - BMDCs	Nrf2-mediated antiviral responses *via* IFN-independent manner - ↓ Replication of SARS-CoV2 and other virus including HSV, VACV, and Zika Virus - ↓ Replication of HSV1 and VACV in type I IFN-deficient cells - ↓ Replication of HSV1 and VACV in IFNAR2 or STAT1-deficient HaCaT cells Anti-inflammatory effects to SARS-CoV2 - ↓ *IFNB1, CXCL10, TNFA*, and *CCL5* in Calu-3 cells - *↓ CXCL10* in PBMCs from healthy donor and patients with severe COVID-19	(56)
Intermediated by ATF3				
**DMI**	LPS	*In vivo*: - Psoriasis mouse model *In vitro*: - BMDMs - BV2 cells - PBMCs - Primary keratinocytes	Activation of electrophilic stress in BMDMs - ↑ Transcriptional markers of Nrf2-dependent responses such as *Hmox1*, *Nqo1* and *Gclm* gene - ↓ LPS-induced the secretion of IL-6, but not TNF in macrophages - ↓ Skin inflammation *in vivo* psoriasis Negative regulation of TLR-mediated secondary, but not primary, transcriptional response *via* ATF/IκBζ signaling pathway - ↓ LPS-induced IL-6**-**IκBζ axis *via* ATF3, but not Nrf2 - ↓ IL-17-mediated IκBζ induction in keratinocytes	(57)
Intermediated by TFEB				
**ITA**	LPS/IFNγ *S.* Typhimurium	*In vivo*: - *Salmonella* infection model *In vitro*: - BMDMs	Antibacterial effects against *Salmonella* Typhimurium infection - ↓ Intracellular growth of *Salmonella* in BMDMs (*In vitro*) or splenic macrophages from mice infected with *S.* Typhimurium SL1344 (*In vivo*) - Activation of Irg1-Rab32–BLOC3 system - Activation of TFEB–Irg1–ITA signaling	(58)
**ITA**	LPS *S.* Typhimurium	*In vivo*: - *Salmonella* infection model *In vitro*: - BMDMs - PBMCs - THP-1 cells	Antibacterial innate immune defense activation - ↑ Lysosomal biogenesis and bacterial clearance *via* TFEB alkylation - ↓ Lethality of mice and inflammation in a murine models of *S.* Typhimurium infection - Activation of IRG1/ITA/TFEB axis	(59)
Intermediated by Akt				
**ITA, DMI, 4-OI**	IAV	Patient sample: - Lung tissue *In vivo*: - IAV model *In vitro*: - PBMCs - THP-1 cells - BMDMs - A549 cells	Anti-inflammatory and –viral functions against IAV infection - ↓ IAV-induced IFN responses in macrophages and human lung tissue explants - ↓ IAV-induced CXCL10 and CCL2 expression - ↓ IAV-induced ROS generation and STAT1 and AKT phosphorylation - ↓ Virion production in A549 and IAV RNA replication in PBMCs - ↓ Pulmonary inflammation and ↑ mice survival in IAV-infected mice	(49)

ITA, itaconate; DMI, Dimethyl itaconate; HCECs, Human corneal epithelial cells; NRF2, Nuclear factor erythroid 2-related factor 2; HO-1, heme oxygenase-1; PMN, Polymorphonuclear neutrophil; LPS, lipopolysaccharide; BMDMs, bone marrow–derived macrophages; NQO-1, Quinone oxidoreductase 1; 4-OI, 4-octyl itaconate; CLP, cecum ligation and puncture; SARS-CoV2, Severe Acute Respiratory Syndrome Coronavirus 2; HSV, Herpes Simplex Virus; VAVV, Vaccinia virus; PBMC, Peripheral Blood Mononuclear cells; BMDCs, Bone marrow-derived dendritic cells; IFNAR2, IFN alpha receptor 2; STAT1, Signal Transducer and Activator of Transcription 1; ATF3, Activating transcription factor 3; TLR, Toll-like receptor; Irg1, Aconitate decarboxylase (Acod1); TFEB, Transcription factor EB; IAV, Influenza A virus; ROS, Reactive oxygen species. ↑ means "increased". ↓ means "decreased".

### 4.1 NRF2 and antioxidant responses

NRF2, a transcriptional factor with a cytoprotective function, is a focus of research on ITA-associated therapeutics for infection and inflammation. The NRF2 protein level and activity are regulated by ubiquitination and degradation by E3 ligase complexes involving Kelch ECH associating protein 1 (KEAP1) (52, 60). However, the underlying regulatory mechanisms are beyond the scope of this review.

ITA-induced alkylation of KEAP1 activates the NRF2 signaling pathway of antioxidant and anti-inflammatory responses (20, 57, 61). Esterified derivatives of ITA, 4-OI, and DMI, are sufficient to activate the NRF2 signaling pathway. For instance, in a model of *Aspergillus fumigatus* keratitis, DMI reduces inflammatory responses in human corneal epithelial cells by activating of NRF2/heme oxygenase (HO)-1 signaling (53). An NRF2 signaling pathway is also important for DMI-mediated anti-inflammatory responses to LPS in macrophages, and DMI induces NRF2, HO-1, and NAD(P)H quinone oxydoreductase 1 (NQO-1), expression, downstream signaling factors of NRF2 signaling (54).

4-OI functions in the resolution of wounds in macrophages. 4-OI suppresses TNF-α, but not IL-6, production *via* NRF2 signaling. 4-OI increases the expression of the immunosuppressive M2 markers TGF-β and CD36, but suppresses collagenase matrix metalloprotease-8 in human monocyte-derived macrophages. In addition, 4-OI alleviates the LPS-induced uptake of fibrous collagen *via* the NRF2 and p38 MAPK signaling pathways (62). In a sepsis model, 4-OI inhibits inflammatory and oxidative stress factors, but increases anti-inflammatory responses, by activating NRF2 signaling (55). Interestingly, 4-OI exerts an antiviral effect against severe acute respiratory syndrome coronavirus-2 (SARS-CoV-2) infection by suppressing host inflammatory responses *via* NRF2 signaling (56). 4-OI and dimethyl fumarate exert antiviral effects against herpes simplex viruses-1 and -2, vaccinia virus, and Zika virus by controlling inflammatory responses (56). Moreover, NRF2 activation suppresses STING expression and signaling, an effect mimicked by NRF2 inducers or 4-OI, to affect STING-dependent inflammatory responses (61). The cyclic guanosine monophosphate-adenosine monophosphate (cGAMP) synthase (cGAS)/STING system is a therapeutic target for IFN-related inflammatory and bacterial infections (61, 63). More data are needed to clarify whether ITA and its family members protect against bacterial infections.

### 4.2 ATF3

ATF3 is a stress-responsive transcription factor of the basic leucine zipper (bZip) family and is essential for controlling physiological functions such as the cell cycle, tumor suppression, and TLR4 signaling (64, 65). An ATF3-mediated signaling pathway regulates the production of inflammatory cytokines, such as IL-6, mediated by ITA and DMI, both of which induce electrophilic stress (57). Whereas TNF is induced by TLR stimulation, IL-6 is produced as a result of secondary transcriptional responses, mainly mediated by IκBζ, which is encoded by *Nfkbiz* (66). Importantly, DMI-mediated ATF3 upregulation suppresses IL-17–mediated IκBζ signaling pathway activation, thus ameliorating skin pathological inflammation *in vitro* and *in vivo* (57). Therefore, the ATF3/IκBζ pathway is a target by which ITA and its derivatives regulate the generation of proinflammatory cytokines.

Mesaconate and citraconic, two isomers of ITA, are immunomodulatory metabolites. They suppress the production of inflammatory cytokines and IFN signaling, and the release of IAV particles from host cells. The anti-inflammatory and antioxidant effects of ITA isomers depend on the NRF2 signaling pathway, and citraconic is the most active NRF2 agonist (67). Mesaconate downregulates glycolysis but does not suppress tricarboxylic acid cycle activity or SDH. Mesaconate significantly reduces the secretion of IL-6 and IL-12 and increases CXCL10 in macrophages. However, this effect is not mediated by NRF2 and ATF3 (68). These data suggest that ITA isomers modulate the NRF2 and ATF3 signaling pathways to influence immune responses in a context-dependent manner.

### 4.3 TFEB

TFEB is a transcription factor of the microphthalmia (MiT/TFE) family (69) that regulates lysosomal biogenesis and autophagy by binding to the CLEAR (coordinated lysosomal expression and regulation) element, which is found in the promoters of lysosomal genes (70, 71). TFEB activation alters carbon funneling to elevate the level of ITA, thereby suppressing *S.* Typhimurium, an intracellular pathogen, in macrophages and *in vivo* (58). Interestingly, *S*. Typhimurium restricts TFEB activity, however, TFEB activation alone is enough to induce *Irg1* and increase the ITA level in macrophages (58). Also, iNOS expression suppresses endogenous ITA synthesis in activated murine macrophages (58). The IRG1-Rab32–BLOC3 pathway is involved in the TFEB-driven ITA transport from mitochondria into *Salmonella*-containing vacuoles to restrict bacterial growth (58). Zhang et al. reported that ITA produced by LPS-stimulated cells induces the alkylation of human TFEB at Cys212, to drive its nuclear translocation and activation, thus suppressing *S*. Typhimurium infection (59). Therefore, the TFEB-associated lysosomal function and ITA synthesis could be leveraged to develop therapeutics for intracellular bacteria.

### 4.4 Akt signaling pathway

Akt/protein kinase B, a downstream serine/threonine protein kinase of phosphoinositide 3-kinase (PI3K), is important in cell growth and survival, cell cycle progression, glucose metabolism, and immune responses (72, 73). Aberrant activation of the Akt pathway contributes to multiple pathological processes during infection, including inflammatory responses, viral propagation (74, 75), and increased intracellular bacterial survival (76, 77). By contrast, the Akt/mTOR-mediated signaling pathway contributes to the non-canonical activation of IFN-dependent antiviral responses (78). IAV-induced pathological inflammation in the lung is increased in IRG1-deficient compared to wild-type mice (49). Importantly, DMI and 4-OI exert the same protective effect as ITA and reduce IFN and inflammatory responses in human PBMCs and lung tissue (49). Mechanistically, both DMI and 4-OI suppress, whereas ITA increases, the phosphorylation of Akt in human monocytic THP1 cells (49). The regulatory effects of ITA and its relatives need to be characterized in terms of Akt signaling modulation and its consequences in viral and bacterial infection.

## 5 Discussion

The roles of ITA and its family members in infection and inflammation have been investigated extensively, but their roles in host defense and pathogenesis during infection are unclear. ITA and its family members exert antimicrobial effects during viral, bacterial, and parasitic infections. However, the IRG1-ITA pathway promotes the pathogenesis of infection in a context-dependent manner. These findings suggest that the complex immunometabolic environment determines the role of IRG1 and ITA in the modulation of host defense against infection. Several pathogens can use ITA as a carbon source during infection. There is no report that esterified derivatives of ITA (4-OI and DMI) are directly used by intracellular pathogens. Therefore, ITA derivatives with similar activities as endogenous ITA show promise as host-directed therapeutics for infectious diseases.

Although the mechanisms by which ITA and its relatives promote host defense against pathogens are unclear, at least four factors—NRF2, ATF3, TFEB, and Akt—are implicated. The esterified forms (4-OI and DMI) are used to surmount the low cell permeability and mimic the functions of ITA. 4-OI inhibits inflammation by alkylating GAPDH and exerts antiviral properties through NRF2 signaling. DMI elicits NRF2 and ATF3 activation in response to bacterial infection, promoting host defense. The two derivatives decreased the phosphorylation of Akt, whereas ITA increases it. Detailed regulation of cellular signaling and comparisons of preclinical and clinical outcomes will further illuminate the unique function of each derivative. It is likely that additional signaling pathways, metabolic remodeling, and factors are involved and should be investigated in greater depth. Further clinical trials are needed to clarify whether ITA and its relatives contribute to antimicrobial or tolerogenic responses during infection. Such efforts will facilitate the development of ITA-based antimicrobials that enhance host immune responses. Overall, the study of ITA and its family members in the context of host defense against infections represents an intriguing area of research with promising implications for the development of novel therapeutic strategies.

## Author contributions

All authors contributed in their order in writing the manuscript. J-MY prepared the table, and E-JP and ISK prepared the figure. All authors wrote this manuscript. E-KJ conceived, wrote this manuscript and is the corresponding author. All authors read and approved the final manuscript.

## Glossary

**Table:**

ACOD1	aconitate decarboxylase 1
ATF3	activating transcription factor 3
BMDM	bone marrow-derived macrophage
bZip	basic leucine zipper
CDK2	cyclin-dependent kinase 2
CF	cystic fibrosis
CFTR	cystic fibrosis transmembrane conductance regulator
cGAMP	cyclic guanosine monophophsate-adenosine monophosphate
cGAS	cGAMP synthase
CLEAR	coordinated lysosomal expression and regulation
CXCL	C-X-C chemokine ligand
DMI	dimethyl itaconate
EPS	extracellular polysaccharides
HO	heme oxygenase
IAV	influenza A virus
IDH2	isocitrate dehydrogenase
IFN	interferon
IL	interleukin
IRG1	immune-responsive gene 1
ITA	itaconate
KEAP1	Kelch ECH associating protein 1
Kp ST258	*Klebsiella pneumoniae* sequence type 258
LPS	lipopolysaccharide
MiT/TFE	microphthalmia
Mtb	*Mycobacterium tuberculosis*
MyD88	myeloid differentiation primary response 88
NF-κB	nuclear factor-kappa B
NQO-1	NAD(P)H quinone oxydoreductase 1
NRF2	nuclear factor erythroid 2-related factor 2
4-OI	4-octyl itaconate
PBMC	peripheral blood mononuclear cell
PI3K	phosphoinositide 3-kinase
PTEN	phosphatase and tensin homolog deleted on chromosome 10
RIPK	receptor interacting protein kinase
ROS	reactive oxygen species
RSV	respiratory syncytial virus
SDH	succinate dehydrogenase
STING	stimulation of interferon genes
TFEB	transcription factor EB
Th1	T helper cell 1
TLR	toll-like receptor
TNF-α	tumor necrosis factor-α.
